# 

**Published:** 1957-01

**Authors:** 


### THE MEDICAL JOURNAL OF THE SOUTH-WEST

*Incorporating*

*The Bristol Medico-Chirurgical Journal*

PUBLISHED UNDER THE AUSPICES OF THE
BRISTOL MEDICO-CHIRURGICAL SOCIETY

*1957 Vol. 72*

J. W. ARROWSMITH LTD., WINTERSTOKE ROAD, BRISTOL

W 1
ME359
v. 72-73
1957-58
c. 1

### 1957 *Contents of Vol. 72*

| | page |
|---|---|
| A Doctor's Visit to U.S.S.R. By A. M. MacLachlan, M.B., CH.B. | 1 |
| The Use and Misuse of Sedatives and Hypnotic Drugs. By R. E. Hemphill, M.A., M.D., D.P.M. | 12 |
| The Public Analyst and the New Food and Drugs Act (1955). By E. G. Whittle, B.SC., F.R.I.C. | 23 |
| Diabetes Mellitus with Renal Papillary Necrosis (Clinical Pathological Conference) | 26 |
| Some Notes of a Medical Visitor to the British West Indies. By A. V. Neale, M.D., F.R.C.P. | 33 |
| From Blue to Pink. By E. G. Bradbeer, M.R.C.S., L.R.C.P., F.F.A.R.C.S., D.A. | 46 |
| The Port Health Service. By D. T. Richards | 56 |
| The Symptoms and Treatment of Acute Follicular Tonsillitis. By S. G. Flavell Matts, M.B. (B'HAM), M.R.C.S. | 63 |
| Astrocytoma Treated by Radiotherapy: Death from Acute Leukaemia (Clinical Pathological Conference) | 67 |
| A Tour of the South-Western Pacific. By A. L. Eyre-Brook, M.S., F.R.C.S. | 75 |
| The Prevention of Acute Rheumatism. By C. Bruce Perry, M.D., F.R.C.P. | 86 |
| Head Injuries. By G. L. Alexander, F.R.C.S. | 91 |
| Bristol and Bordeaux. By J. A. Cosh, M.D., B.CHIR., M.R.C.P. | 98 |
| Sarcoidosis Treated with Cortisone (Clinical Pathological Conference) | 101 |
| Clinical Pathology and General Practice. By H. J. Gibson, M.D., D.P.H. | 109 |
| Towards Better Health at Work. By T. G. Faulkner Hudson, M.D., M.R.C.P. | 117 |
| Child Art. By D. E. Milner, O.B.E., A.R.C.A., R.W.A. | 125 |
| Acute Necrosis of the Liver in Pregnancy (Clinical Pathological Conference) | 129 |
| Correspondence | 29 |
| Notes and News | 29, 73, 106 |
| Appointments | 30, 73, 107, 145 |
| Members of the Bristol Medico-Chirurgical Society | 133 |
| Subscribers to the Journal (Non-Members) | 140 |
| News from Societies | 141 |

33375.27

THE

MEDICAL JOURNAL OF THE SOUTH-WEST

*(Incorporating the Bristol Medico-Chirurgical Journal)*

Established 1883

VOL. 72 (i) January, 1957 NO. 263

### Contents

| | | Page |
|---|---|---|
| A DOCTOR'S VISIT TO U.S.S.R. . . | *Dr. A. M. MacLachlan* | 1 |
| THE USE AND MISUSE OF SEDATIVES AND HYPNOTIC DRUGS | *Dr. R. E. Hemphill* | 12 |
| THE PUBLIC ANALYST AND THE NEW FOOD AND DRUGS ACT (1955) . . . . . . | *Mr. E. G. Whittle* | 23 |
| DIABETES MELLITUS WITH RENAL PAPILLARY NECROSIS | *Clinical Pathological Conference* | 26 |
| CORRESPONDENCE . . . . . . . | | 29 |
| NOTES AND NEWS . . . . . . . | | 29 |
| APPOINTMENTS . . . . . . . . | | 30 |

*PUBLISHED QUARTERLY*

*ANNUAL SUBSCRIPTION* **16/-** *POST FREE*

PUBLISHED UNDER THE AUSPICES OF THE
BRISTOL MEDICO-CHIRURGICAL SOCIETY

### *Editorial Committee*

Dr. J. E. Cates, Department of Medicine, Bristol Royal Infirmary. *Editor.*

Dr. N. J. Brown, Southmead Hospital, Bristol. *Assistant Editor.*

Dr. J. Macrae, Ham Green Hospital.

Dr. R. C. Wofinden, Central Clinic, Bristol.

Mr. A. E. S. Roberts, Medical Library, University of Bristol. *Secretary.*

### *Local Correspondents*

*Bath* . . Mr. A. D. Bateman, 3 The Circus, Bath.

*Exeter* . . Dr. L. N. Jackson, Mount Jocelyn, Crediton, Devon.

*Gloucester* . Dr. R. F. Jarrett, 9 College Green, Gloucester.

*Plymouth* . Dr. C. R. Croft, Lexden, Hartley Av., Plymouth.

*Taunton* . Dr. M. McAlley, 1 The Crescent, Taunton.

*Torquay* . Dr. A. Robinson Thomas, 20 Devon Square, Newton Abbot, Devon.

*Truro* . . Dr. F. D. M. Hocking, St. Andrews, Perranporth, Cornwall.

*Yeovil* . . Mr. J. A. Vere Nicoll, Knapp House, Yeovil, Somerset.

### NOTICE TO CONTRIBUTORS

Original articles are invited, provided they have not been published elsewhere. Notes of forthcoming events, meetings held, degrees conferred, staff appointments, and other local news are welcomed. The editorial committee reserves the right to make literary corrections.

Illustrations should always be supplied ready for reproduction. All re-touchings or other operations required will be charged to the author. Line drawings should be drawn in Indian ink. We regret that we must ask authors to pay for making blocks, if this is necessary.

J. W. ARROWSMITH LTD., WINTERSTOKE ROAD,
BRISTOL.

### Correspondence

### Notes and News

Professor C. Bruce Perry has been appointed to the panel of experts on " Chronic and Degenerative Diseases" of the World Health Organisation for the period 1956–61.

Professor G. A. Smart gave the Honyman Gillespie Lecture on "The Nephrotic Syndrome" at the University of Edinburgh on 8th November, 1956.

Dr. Percy Stocks was presented with the Jenner Medal at a meeting of the Section of Epidemiology and Preventive Medicine of the Royal Society of Medicine on 16th November, 1956.

Dr. I. McD. G. Stewart was awarded the Buckston Browne Prize for 1956 for his essay on hypertension.

*M.R.C.P.:* Margaret H. Buston, M.B., Ch.B., Bristol.

*New Year Honours:*

Baron: Sir Robert Sinclair, for public services.

Knight Bachelor: Major Egbert Cadbury, for public services in Somerset and Gloucestershire.

*Obituary:* Dr. Dorothy Woodman.

### UNIVERSITY OF BRISTOL

### *EXAMINATION RESULTS*

*Doctor of Medicine:* Lisa E. Hill, R. Hill, D. R. Coles, C. H. Gurd.

*Ph.D.* (in the Faculty of Medicine): S. M. A. Zaidi.

*M.B., Ch.B.:*

B. H. Burns

J. H. Chick

I. E. Doney

J. B. Griffiths

H. G. Handel

C. Hemming

R. W. Hiles

Evelyn B. Horton

S. C. Jordan

D. J. Mahy

J. Martyrossian

Penelope Neville

W. D. L. Revill

G. B. Simon

Sara Waters

T. C. Waters

J. S. A. Ashley

Edna M. Dumbell

M. J. Lewis

R. D. Neale

Hilary S. Prowse

W. B. Whisker

### *Appointments*

### *UNITED BRISTOL HOSPITALS*

*November* 1956 *to February* 1957

| *Name* | *Appointment* | *Formerly* |
|---|---|---|
| MALCOMSON, K. G., M.B., B.CH., B.A.O. (QUEEN'S UNIV. BELFAST, F.R.C.S. (ENG.), D.L.O. (LOND.). | Consultant, Ear, Nose and Throat Surgery. | Senior E.N.T. Registrar, Cardiff Royal Infirmary. |
| DALTON, G. A., M.B., CH.B., F.R.C.S., D.L.O. | Senior Registrar in Ear, Nose and Throat Surgery. | Registrar in Ear, Nose an[illegible] Throat Surgery, United Birmingham Hospitals. |
| STEPHENS, MISS E., M.B., CH.B. | Registrar in Dermatology. | S.H.O. in Dermatology. |
| KAPUR, Y. P., M.B., B.S. (PUNJAB). | Registrar in Ear, Nose and Throat Surgery. | Chief Resident, Ear, Nose [illegible] Throat Department, Bo[illegible] City Hospital, U.S.A. |
| CHORLEY, G. E., M.B., B.S., D.A. | Registrar in Anaesthetics. | S.H.O. in Anaesthetics. |
| GILES, R., M.B., B.S. | Registrar in Anaesthetics. | S.H.O. in Anaesthetics, Middlesex Hospital. |

### *SOUTH-WESTERN REGIONAL HOSPITAL BOARD*

*November* 1956 *to January* 1957

### *BRISTOL*

| *Name* | *Appointment* | *Formerly* |
|---|---|---|
| McKENNA, J. J., L.D.S. (R.F.P.S.). | Registrar to the Maxillo-Facial Unit at Frenchay Hospital and in Bristol Dental Hospital. | S.H.O., Maxillo-Facial Un[illegible] Withington Hospital, Manchester. |
| WILSON, M. G., M.B., CH.B. (LEEDS), F.R.C.S. | Consultant Surgeon, Southmead Hospital, Bristol. | Nuffield Research Fellow [illegible] Surgery, University of Bristol. |
| WOODS, GRACE E., M.B., B.S. (LOND.), D.P.H., D.C.H., M.D. (BRISTOL). | Deputy Medical Superintendent, Hortham Hospital, Almondsbury, near Bristol. | Part-time appointment in Bristol Public Health De[illegible] ment, Clinical Assistant, Bristol Children's Hos[illegible] |
| YOUSSEF, M. K. A., M.B., CH.B. (CAIRO). | Registrar, Winford Orthopaedic Hospital (joint appointment with the United Bristol Hospitals). | S.H.O. (General Surgery), Ingham Infirmary, Sout[illegible] Shields. |
| KING, MARGUERITE A. R., M.B., CH.B. (BRISTOL). | Registrar to Department of Pathology, Frenchay Hospital. | Registrar in Clinical Patho[illegible] University College Hos[illegible] Ibadan, Nigeria. |

### *NORTH GLOUCESTERSHIRE*

| | | |
|---|---|---|
| LOMAS, J., M.B., B.CHIR., D.P.M. (R.C.S. AND P.). | Consultant Psychiatrist, Horton Road and Coney Hill Hospitals, Gloucester. | Assistant Psychiatrist, Sp[illegible] field Hospital, London. |

### *BATH*

| | | |
|---|---|---|
| RUSSELL, J. B. G., M.B., CH.B. (ABERDEEN), M.R.C.P. (LONDON), D.P.M. (ENG.). | Medical Superintendent, Roundway Hospital, Devizes. | Deputy Medical Superi[illegible] dent, Tone Vale Hos[illegible] near Taunton. |
| STEWART, D. J., M.B., CH.B. (GLAS.). | Clinical Assistant in Orthopaedic and Traumatic Surgery, Royal United Hospital, Bath. | — |

### SOUTH SOMERSET

| *Name* | *Appointment* | *Formerly* |
|---|---|---|
| NIGHTS, L. E. D., M.B., B.S. (LONDON), F.R.C.S. (ENG.). | Consultant Surgeon, Yeovil District Hospital (one session weekly). | Also S.H.M.O. in General Surgery, The Yeatman Hospital, Sherborne. |

### DEVON AND CORNWALL

| *Name* | *Appointment* | *Formerly* |
|---|---|---|
| YDE, M. T., M.B., B.CH. (N.U.I.). | Anaesthetic Registrar, South Devon and East Cornwall Hospital, Plymouth. | Anaesthetist, Eye, Ear, Nose and Throat Hospital, Cork. |
| ZAM, M. A., M.B., B.S. (PUNJAB), F.R.C.S. (EDIN.). | Surgical Registrar, South Devon and East Cornwall Hospital, Plymouth. | In Department of Surgery, Monmouth Memorial Hospital, New Jersey, U.S.A. |
| AILANI, T. I., M.B., CH.B. (BAGHDAD), F.R.C.S. (EDIN.). | Surgical Registrar, South Devon and East Cornwall Hospital, Plymouth. | — |
| RANKLIN, R. T. D., M.B., CH.B. (LEEDS), D.P.M. | Assistant Psychiatrist, Digby-Wonford Hospital, Exeter. | J.H.M.O., Banstead Hospital, Sutton, Surrey, and Clinical Assistant in Department of Psychological Medicine, Westminster Hospital. |
| RINGLE, E. P., M.B., B.CH. (DUBLIN), D.P.M. | Senior Psychiatric Registrar, Moorhaven Hospital, Ivybridge. | Locum tenens S.H.M.O., Broadgate Hospital, Beverley, Yorks. |
| EWIS, E. C., M.B., B.S. (LOND.). | Registrar, Mount Gold Orthopaedic Hospital, Plymouth. | — |
| TONEMAN, M. E. R., M.B., CH.B. (MANCHESTER), D.C.H. | Registrar in Paediatrics, Royal Devon and Exeter Hospital, Exeter. | S.H.O. in Paediatrics, Royal Devon and Exeter Hospital, Exeter. |

